# Defined Daily Dose and Appropriateness of Clinical Application: The Coxibs and Traditional Nonsteroidal Anti-Inflammatory Drugs for Postoperative Orthopaedics Pain Control in a Private Hospital in Malaysia

**DOI:** 10.3390/pharmacy8040235

**Published:** 2020-12-08

**Authors:** Faizah Safina Bakrin, Mohd Makmor-Bakry, Wan Hazmy Che Hon, Shafeeq Mohd Faizal, Mohamed Mansor Manan, Long Chiau Ming

**Affiliations:** 1 School of Pharmacy, KPJ Healthcare University College, Nilai 71800, Negeri Sembilan, Malaysia; 2 Faculty of Pharmacy, Universiti Kebangsaaan Malaysia, Kuala Lumpur 50300, Malaysia; mohdclinpharm@ukm.edu.my; 3 School of Medicine, KPJ Healthcare University College, Nilai 71800, Negeri Sembilan, Malaysia; whazmy@hotmail.com; 4 KPJ Seremban Specialist Hospital, Seremban 70200, Negeri Sembilan, Malaysia; shafeeq@kpjseremban.com; 5 Faculty of Pharmacy, Universiti Teknologi MARA, Puncak Alam 42300, Selangor, Malaysia; mmmanan2002@yahoo.com; 6 PAPRSB Institute of Health Sciences, Universiti Brunei Darussalam, Gadong BE1410, Brunei; longchiauming@gmail.com

**Keywords:** pharmacoepidemiology, prescribing pattern, drug utilization, tNSAIDs, coxibs

## Abstract

Introduction: Drug utilization of analgesics in a private healthcare setting is useful to examine their prescribing patterns, especially the newer injectable cyclooxygenase (COX)-2 inhibitors (coxibs). Objectives: To evaluate the utilization of coxibs and traditional nonsteroidal anti-inflammatory drugs (tNSAIDs) indicated for postoperative orthopaedic pain control using defined daily dose (DDD) and ratio of use density to use rate (UD/UR). Method: A retrospective drug utilization review (DUR) of nonsteroidal anti-inflammatory drugs (NSAIDs) at an inpatient department of a private teaching hospital in Seremban, Malaysia was conducted. Patients’ demographic characteristics, medications prescribed, clinical lab results, visual analogue scale (VAS) pain scores and length of hospital stay were documented. Orthopaedic surgeries, namely arthroscopy, reconstructive, and fracture fixation, were included. Stratified random sampling was used to select patients. Data were collected through patients’ medical records. The DDD per 100 admissions and the indicator UD/UR were calculated with the World Health Organization’s DDD as a benchmark. The inclusion criteria were patients undergoing orthopaedic surgery prescribed with coxibs (celecoxib capsules, etoricoxib tablets, parecoxib injections) and tNSAIDs (dexketoprofen injections, diclofenac sodium tablets). Data were analysed descriptively. This research was approved by the academic institution and the hospital research ethics committee. Result: A total of 195 records of patients who received NSAIDs were randomly selected among 1169 cases. In term of the types of orthopaedic surgery, the ratio of included records for arthroscopy:fracture fixation:reconstructive surgery was 55.4:35.9:8.7. Most of the inpatients had low rates of common comorbidities such as cardiovascular disease as supported by their baseline parameters. The majority were not prescribed with other concomitant prescriptions that could cause drug interaction (74.9%), or gastroprotective agents (77.4%). Overall, DDDs per 100 admissions for all NSAIDs were less than 100, except for parecoxib injections (389.23). The UD/UR for all NSAIDs were less than 100, except for etoricoxib tablets (105.75) and parecoxib injections (108.00). Discussion: As per guidelines, the majority (96.9%) received other analgesics to ensure a multimodal approach was carried out to control pain. From the UD/UR results, the arthroscopy surgery was probably the most appropriate in terms of NSAID utilization. Conclusion: The prescribing pattern of NSAIDs except parecoxib was appropriate based on adverse effect and concurrent medication profile. The findings of this DUR provide insight for a low-risk patient population at a private specialized teaching hospital on the recommended use of NSAIDs for postoperative orthopaedic pain control.

## 1. Introduction

There has been an increase in the number of patients in almost all surgical disciplines in Malaysia, and orthopaedic surgery was ranked second highest after general surgery in terms of the number of inpatients [1]. Orthopaedic surgery can be categorized by surgical techniques (i.e., fixation, bone grafting and arthroscopy) that are related to musculoskeletal disorders affecting muscles, ligaments, tendons, and bones of various parts of the body such as the shoulder, hip, leg and hand. Most patients will experience pain after surgery and the pain should be alleviated as soon and as effectively as possible to promote healing and prevent potential complications. The literature shows that, among 179 surgical groups at 105 German hospitals, patients that underwent 22 orthopaedic procedures demonstrated a high pain intensity on the first day after surgery [2]. Hence, healthcare providers are urged to monitor patients’ pain regularly during hospital admission and treat patients based on their pain experience [3]. Postoperative pain is categorized as acute pain that should be managed in a multimodal approach, which consists of utilization of multiple analgesics, including opioids, non-opioids and other adjuvants [4,5].

In patients with no contraindications, nonsteroidal anti-inflammatory drugs (NSAIDs), including both traditional nonselective NSAIDs (tNSAIDs) and the selective cyclooxygenase (COX)-2 inhibitors (coxibs), are the drugs of choice for surgery analgesic regimes [4]. Both tNSAIDs and coxibs are widely utilized for orthopaedic postoperative pain management [6,7]. The most recent review study found that both NSAIDs and coxibs increased patient satisfaction and decreased opioid use, so were concluded to be safe and effective in the treatment of postoperative pain including orthopaedic surgery [8].

In Malaysia, the healthcare system is characterised by a two-tier system, which consists of the public and private healthcare sectors. The public healthcare sector system is funded by the government, and the Ministry of Health (MOH), as the largest healthcare service provider, controls and procures medicines at lower prices compared to the private healthcare sector. The private healthcare settings in Malaysia can essentially be divided into three: (a) general practitioner clinics, (b) community pharmacies, and (c) private hospitals. The private sector provides a healthcare service for a non-subsidised fee-for-service through a large network of private general practitioner clinics, community pharmacies and hospitals financed through out-of-pocket payments, private insurance or third-party payments [1,9,10].

According to Malaysian Statistics on Medicines 2011–2014 [9], utilization of NSAIDs and antirheumatic drugs (ATC: M01A) in the private sectors were more than double that of the public sectors. The private sector information was derived from sales data from pharmaceutical companies and vendors collected through Quintiles IMS Malaysia. In the private sector report, expenditure on anti-inflammatory and antirheumatic drugs was ranked as sixth among the top ten drugs by expenditure in 2014 with a total expenditure of MYR 111,230,000 (equivalent to USD 26,775,964) and an estimated 0.96% of the population (9.6405 defined daily dose (DDD)/1000 population/day) utilized these medicines according to therapeutic group. However, information derived from sales data alone is not enough to help the management of private sectors to understand, interpret, evaluate, and improve the prescribing, administration and use of NSAIDs. This study was conducted at a main branch of the largest private hospital group in Malaysia, which has a similar setting to other private hospitals in the country. The findings are very applicable in terms of projecting the use of tNSAIDs and coxibs. Thus, the primary objective of this study was to evaluate the utilization of coxibs and traditional nonsteroidal anti-inflammatory drugs (tNSAIDs) indicated for postoperative orthopaedic pain control in a private hospital located in the central part of Malaysia. The utilization was analysed using defined daily dose (DDD) and ratio of use density to use rate (UD/UR).

## 2. Methods

This preliminary retrospective drug utilization review of NSAIDs for postoperative orthopaedic inpatients was conducted in a private teaching hospital with 188 bed multidisciplinary ward services. The overall information of orthopaedic surgery from January 2018 to December 2018 was screened. A total of 1169 orthopaedic surgeries were listed and categorized into 76 types of surgeries. This study focused on three types of major orthopaedic surgeries: category 1: arthroscopy (examples: acromioplasty, anterior cruciate ligament); category 2: reconstructive surgery (examples: total knee replacement, hemiarthroplasty); category 3: fracture fixation (examples: close manipulative reduction, dynamic hip screw) (Supplementary Table S1). A sample size of 195 patients was determined using a sample size calculator available online (http://www.raosoft.com/samplesize.html). The records were stratified proportionally to the three categories of surgeries. Samples were selected using a random number generator in Excel (Microsoft Office Professional Plus 2016). The inclusion criteria were warded patients undergoing orthopaedic surgery at the selected hospital who were prescribed coxibs or tNSAIDs. Exclusion criteria were patient profiles with missing data [11], under 18 years of age and non-Malaysian patients. Here missing data were defined as when there was no oral or injectable tNSAIDs or coxibs logged in a patient’s record. However, for data that were missing completely at random [12], such as baseline parameters, we included the patients for the purpose of this drug utilization review (DUR) study.

After screening for patient registration numbers from the operation theatre’s database, patients′ medical folders together with the intranet system-based medical records were accessed to retrieve the following data:

- Age, gender, ethnicity of the patient;
- Length of stay and hospital discharge;
- Baseline parameters: serum creatinine (µmol/L), serum glucose (mmol/L), LDL-cholesterol (mmol/L), total cholesterol (mmol/L) and C-reactive protein (mg/L), measured on the first day upon admission before surgery;
- Visual analogue scale (VAS) pain score;
- List of tNSAIDs and/or coxibs and their maintenance dose per day;
- Types of other analgesics prescribed for patient;
- Concomitant prescriptions of:
  - Gastroprotective agent (GPA) (e.g., proton pump inhibitor (PPI), histamine-2 receptor antagonist (H2RA), or prostaglandin E1 analogue;
  - Drugs that may cause drug interaction (e.g., diuretics, beta blockers and ACE inhibitors) with tNSAIDs and/or coxibs;
  - Possible adverse effects.

The visual analogue scale (VAS) pain score was recorded in the nursing VAS chart after admission (preoperative) and during the post-surgical ward rounds, on average 2–3 times daily. Concomitant prescriptions were taken from the intranet system for accurate records. The adverse effects related to NSAIDs were determined by searching patients′ records for any incidence of myocardial infarction, stroke, or gastrointestinal symptoms.

This study was approved by two institutional review boards (IRBs); first, the academic research institution (KPJ Healthcare University College Research Committee and Research Ethics Committee), and second, the hospital (KPJ Seremban Specialist Hospital Research and Quality Innovation Committee). As per the teaching hospital’s policy, every patient signed the informed consent related to patient data confidentiality and compliance with the Declaration of Helsinki, allowing the review of their medical records for the purpose of education, management improvement and medical advancement.

## 3. Data Analysis

Data analysis was performed using SPSS (version 23.0). The distribution of each categorical variable was examined by an analysis of frequencies and percentages. The normality test of each numerical variable was evaluated by the Shapiro–Wilk test. For the normal variables, mean and standard deviation (mean ± SD) were used. For non-normal variables, median and interquartile range (IQR) as a range were used.

The NSAIDs, including tNSAIDs and coxibs that had been identified in this study, were classified according to the anatomical therapeutic chemical (ATC) classification (Supplementary Table S2). All DDDs for drugs in this study were searched for and retrieved using the WHO Collaborating Centre for Drug Statistics Methodology: diclofenac sodium tablet (DDD_WHO_ = 100mg); parecoxib injection (DDD_WHO_ = 40 mg); etoricoxib tablet (DDD_WHO_ = 60 mg) and dexketoprofen injection (DDD_WHO_ = 75 mg) [13].

Data reflected by DDD on drug consumption only present a basic estimation on the consumption of the drug usage instead of the actual utilization. Researchers can compare between patients’ groups and evaluate patterns in the consumption of drugs as DDD has a fixed unit of measuring the dose regardless of the dosage form (strength of the tablet) and price.

DDDs per 100 admissions was used instead of DDDs per bed days because the hospital in this study provides a multidisciplinary ward service. DDDs per 100 admissions estimate the percentage of patients admitted for orthopaedic surgery that may be prescribed one DDD of a NSAID for every day in that year. Its formula is:

(1) $$DDDs\;per\;100\;admissions\;=\;\frac{number\;of\;DDDs\;per\;year\;\left(DDD_{drug/year}\right)\;\times \;100\;patients}{number\;of\;admissions}$$

The numerator of the above formula, “number of DDDs per year” (DDD_drug/year_), is the total one-year usage of the specific drug, with the strength of the drug in the same unit as the DDDWHO. Its formula is:

(2) $$Number\;of\;DDDs\;per\;year\;\left(DDD_{drug/year}\right)\;=\;\frac{total\;drug\;prescribed\;for\;adult\;patient\;\left(Drug\;prescribed\right)}{DDD\;level\;assigned\;by\;the\;WHO\;\left(DDD_{WHO}\right)}$$

The ratio of use density to use rate (UD/UR) was also calculated to evaluate the appropriateness of clinical application [14] of both tNSAIDs and coxibs, by adapting the formula below:

(3) $$UD/UR\;=\;\frac{number\;of\;DDDs\;per\;year\;\left(DDD_{drug/year}\right)\;\times \;100}{number\;of\;inpatients\;receiving\;NSAID\;\times \;average\;length\;of\;stay}$$

The UD/UR has been found to be correlated with the drug utilization index (DUI), where a DUI of more than 1.0 indicates a possibility of overdose and a DUI less than or equal to 1.0 indicates that the dose is rational [14]. This suggests that a UD/UR of more than 100 indicates a possibility of irrational medication and a UD/UR less than or equal to 100 indicates a possibility of rational medication.

## 4. Result

A total of 195 case records were reviewed: 132 males and 63 females. The majority of the patients (89.8%) were in the age group of less than 60 years old, and the highest age was 94. Among the selected three major types of orthopaedic surgeries, arthroscopy was the highest proportion (55.4%), followed by fracture fixation (35.9%) and reconstructive surgery (8.7%). For numerical variables, all non-normal distributed data, except total cholesterol, are reported in median (Table 1).

During admission, there were other medications that were prescribed to patients (Table 2). Most of the patients had not been prescribed with any gastroprotective agent (77.4%) nor any medication for comorbid condition (74.9%).

The utilization of tNSAIDs and coxibs prescriptions during admission are categorized as single and combinations of same or different types of NSAIDs (Table 3). The majority of patients (64.1%) received one COX-2 inhibitor either orally or via injection.

DDD indicators and their related variables among types of surgeries are shown in Table 4 and the interpretation is clarified in Supplementary Table S3. Overall, DDDs per 100 admissions for all NSAIDs were less than 100, except for parecoxib injections (389.23). The UD/UR for all NSAIDs were less than 100, except for etoricoxib tablets (105.75) and parecoxib injections (108.00).

## 5. Discussion

Sales data of each drug are not sufficient to generate information to understand, interpret, evaluate and improve the prescribing, administration and use of NSAIDs. Therefore, the drug utilization data will be useful for the pharmacy as well as management team for auditing and quality improvement.

### 5.1. Patient Demographic and Characteristic

The findings from this study based on the baseline parameters suggest that most of the patients had low rates of most common comorbid conditions such as cardiovascular events and underlying chronic renal disease. Previous studies have suggested that private specialized hospitals provide treatment to lower-risk patient populations than competing general hospitals [15,16]. This is also supported by the findings from this study that revealed that, during admission, most of the patients (74.9%) had not been prescribed any other drugs that may cause drug interaction with NSAIDs (Table 2). Gastroprotective agents are added to the NSAIDs regimen as one method of reducing adverse gastrointestinal events associated with NSAIDs [17]. The majority of patients were not prescribed any gastroparetic agents during admission (151 out of 195, 77.4%) or follow-up (186 out of 189, 98.4%), which suggests that patients might be at low risk of gastrointestinal adverse outcomes. The duration of follow-up on any prescribed gastroparetic agents was up to two months, to avoid any adverse gastrointestinal outcomes [18].

### 5.2. Multimodal Analgesia

As presented in Table 2, the majority of the patients received other analgesics through more than 80 different combinations that may be customised accordingly, with the aim of achieving the desired level of pain relief. These combinations, wherein drugs from the opioid and non-opioid group are given synergistically, adhere to Malaysia Ministry of Health guidelines [4,5]. The other analgesics identified in this study were strong opioids (fentanyl injections, morphine injections, pethidine injections, oxycodone injections), weak opioids (tramadol injections and capsules), paracetamol (injections and tablets), a combination of paracetamol–codeine tablets, a combination of paracetamol–tramadol tablets, muscle relaxants (eperisone tablets, Esmeron injections), anxiolytics (alprazolam tablets, diazepam tablets), antidepressants (amitriptyline tablets) and anticonvulsants (gabapentin tablets). This procedure for managing postoperative pain is called “multimodal analgesia”, consisting of the use of a variety of analgesic medications and different routes that target different mechanisms of action in the peripheral and/or central nervous system [19]. Opioids have been the conventional treatment for postoperative pain. However, their actions are limited to providing analgesia to the central nervous system sites; they do not act against the inflammatory component of pain. The majority of patients (184 out of 195, 94.4%) were given at least one opioid for severe pain, though a few recommendations suggest using NSAIDs to manage postoperative pain and minimize opioid-induced adverse events [8,19,20,21].

### 5.3. Overall NSAIDs Utilization

During admission (Table 3), some patients (60/195, 30.8%) received more than one NSAID, which may be due to insufficient analgesia with a single agent [22]. It was revealed that the coxibs were prescribed more than the tNSAIDs (Table 3) and we calculated the DDD indicators (Table 4). The utilization of tNSAIDs was low for dexketoprofen and diclofenac, 10.60 DDD/100 admissions and 2.05 DDD/100 admissions, respectively. The UD/UR for both tNSAIDs covered in this study were less than 100, indicating the appropriateness of clinical application. Other than established evidence on complicated gastrointestinal events of tNSAIDs [5,23] that may affect patients′ adherence, there is no evidence to determine that any of the coxibs are more efficient for postoperative pain control than the tNSAIDs. The often-cited reasons for the preference of coxibs are the reduced incidence of adverse gastrointestinal effects, patient preferences and cost-effectiveness [24,25]. Most of the patients were covered by insurance (183/195, 93.8%), which may have influenced the prescribers to choose the coxibs instead of tNSAIDs, as compared to public hospitals [10].

Overall, the rate of prescribed parecoxib usage was the highest based on the number of DDDs per year (759) and DDDs per 100 admissions (389.23). This was similar to the trend of parecoxib utilization in Malaysia that has shown the highest increase and was mostly contributed by the private sector [9]. Parecoxib was reported better than tNSAIDs for postoperative analgesia with minimal adverse effects [26,27]. The UD/UR for parecoxib injection in total was 108.00, suggesting that the utilization of parecoxib may require further close monitoring by the clinical audit team. However, no adverse effects related to parecoxib were reported in this study. This finding can be confirmed with the systematic review that concluded the safety profile of parecoxib to be utilized for managing postoperative pain [28]. A clinical trial has suggested that a lower dose administration of parecoxib 20 mg, once or twice daily, was also sufficient for effective postoperative pain control [29]. This strategy may be useful in achieving the rational use of parecoxib.

With regard to the analgesic effect over 12 h, parenteral parecoxib 40 mg given before surgery was reported as more effective than oral celecoxib 400 mg given after surgery [30]. This was probably the reason for why parecoxib was preferred to celecoxib among the study site’s prescribers, where only 67.2% of patients (Table 4) were prescribed with celecoxib upon admission for orthopaedic surgery. The wide usage of celecoxib is confirmed with the national data in which celecoxib was ranked third among all NSAIDs used after diclofenac and mefenamic acid [9]. Some studies have reported that celecoxib was effective for perioperative management in reducing pain and total opioid use with minimal adverse effects [30,31,32]. The UD/UR for the celecoxib capsule was 81.73 in total, suggesting an appropriate clinical application with dosage given in accordance with WHO′s DDDs (Table 4). In this study, the 400 mg celecoxib dose once daily or 200 mg celecoxib dose twice daily was noted in the database complied with guidelines (MIMS Malaysia). Some studies have reported that celecoxib was better than tNSAIDs in terms of safety profile and pain control effectiveness [19,33].

Overall, the utilization of the etoricoxib tablet was 57.95 DDD/100 admissions; but the UD/UR was 105.75 (Table 4), indicating that closer monitoring is needed. However, there were no adverse effects related to etoricoxib reported in this study. This finding can be confirmed with the systematic review that concluded the safety profile of etoricoxib and its effectiveness to be utilized for managing postoperative pain [34,35,36]. In this study, 60 mg, 90 mg, or 120 mg etoricoxib dose once daily was noted in the database, which are compliant with the guidelines (MIMS Malaysia). A systematic review has revealed that a single 90 mg dose of etoricoxib was as effective as a single 120 mg dose of etoricoxib in producing useful pain relief for 20 h in half of the people treated [34]. This strategy may be useful in achieving the rational use of etoricoxib.

For the overall utilization of NSAIDs in the study site, there were a few factors mentioned above that influenced prescribers to select coxibs instead of tNSAIDs. To sum up, coxibs are preferable because of their pharmacology profile, i.e., long duration of action, reduced incidence of tNSAIDs-related gastrointestinal side effects, and consequent reduced opioid use and related side effects [22,37].

### 5.4. NSAIDs Utilization in Each Type of Surgery

We calculated the DDD indicators of all NSAIDs in each type of surgery (Table 4). In all types, utilization of both tNSAIDs (dexketoprofen, diclofenac) were less than 100 for both DDDs/100 admissions and UD/UR, excluding dexketoprofen, which was not utilized in reconstructive surgery. Arthroscopy surgery demonstrated that all utilized NSAIDs (except parecoxib injections) were less than 100 for both DDDs/100 admissions and UD/UR. There was a similar pattern with arthroscopy surgery: the DDDs/100 admissions in fixation surgery for all utilized NSAIDs (except parecoxib injections) were less than 100. However, the UD/UR in fixation surgery indicated more than 100 for both parecoxib and etoricoxib, suggesting the inappropriateness of clinical application and the need for enhanced monitoring. Meanwhile, for reconstructive surgery, the DDDs per 100 admissions for all coxibs (celecoxib capsules, etoricoxib tablets, parecoxib injections) were more than 100. Contrary to common perception [38], the UD/UR for parecoxib and etoricoxib in reconstructive surgery were 73.26 and 62.50, respectively, suggesting the appropriateness of clinical application. Arthroscopy, reconstructive and fixation surgeries demonstrated high-pain intensity on the first day after the surgery [2]. This phenomenon was confirmed in this study, where higher use of parecoxib and etoricoxib is evident. Another study is also in agreement with this notion because orthopaedic surgeons prescribed more NSAIDs than other specialists—gynaecologists, paediatricians, general surgeons, internists and dentists [39]. Generally, arthroscopy procedures are less complicated than reconstructive and fracture fixations [40,41]. This might be the reason why the utilization of NSAIDs was appropriate in arthroscopy surgery.

## 6. Conclusions

Overall, the utilization of NSAIDs at the selected site of study was appropriate, but some NSAID dosages given were not in accordance with the WHO′s DDDs. As per the guidelines, almost all records showed patients received other analgesics to ensure a multimodal approach was carried out to control pain. Overall, DDDs per 100 admissions for all NSAIDs resulted in less than 100 except for parecoxib injections. The UD/UR for all NSAIDs were less than 100 except for etoricoxib tablets and parecoxib injections. Gastroprotective agents, as per recommendation by clinical practice guidelines, were not prescribed unnecessarily. Arthroscopy surgery demonstrated the most appropriate utilization of NSAIDs. The findings of this DUR provide an insight for a low-risk patient population at a private specialized teaching hospital. It is essential to perform the NSAIDs DDD indicators calculation individually for each type of surgery, for better monitoring purposes.

## Figures and Tables

**Table 1:** Patient demographics and characteristics.

Characteristics	*n*	Mean ± SD */Median (IQR) **
Age (year)	195	40 (28–53) **
Pain score before surgery (maximum pain score of ten)	195	3 (2–3) **
Pain score during admission (maximum pain score of ten)	195	3.5 (3.0–4.5) **
Length of stay in hospital after surgery (day)	195	3 (2–4) **
Total length of stay in hospital (day)	195	4 (3–5) **
Baseline parameters		
Serum creatinine (µmol/L) *[normal range 51–115]*	161	78 (60–91) **
Serum glucose (mmol/L) *[normal range 3.9–6.1]*	148	5.8 (5.1–7.4) **
LDL-cholesterol (mmol/L) *[risk indicator > 4.1]*	146	3.0 (2.3–3.7) **
Total cholesterol (mmol/L) *[risk indicator > 6.2]*	146	5.2 ± 1.0 *
C-reactive protein (mg/L) *[<1 mg/L (Low risk), 1–3 mg/L (Average risk),* *>3 mg/L (high risk)]*	139	2.6 (1.5–8.4) **

Note: * normal numerical variable; ** non-normal numerical variable. Abbreviation: SD, standard deviation; IQR, interquartile range.

**Table 2:** Concomitant prescribed medication at admission.

	*n*	%
Gastroprotective Agent		
None	151	77.4
One Proton Pump Inhibitor	40	20.5
One Histamine-2 Receptor Antagonist	1	0.5
One prostaglandin E1 analogue	1	0.5
Two Proton Pump Inhibitors	2	1.0
Medication for Comorbid Condition		
None	146	74.9
One medication	25	12.8
Anticoagulant alone	6	
Diabetes alone	6	
Hypertension alone	6	
Asthma alone	3	
Heartburn alone	2	
Gout alone	2	
Hyperlipidaemia alone	0	
Medication for Comorbid Condition		
Two medications	10	5.1
Hypertension + Hyperlipidaemia	3	
Anticoagulant + Hypertension	2	
Asthma + Hypertension	1	
Asthma + Diabetic	1	
Gout + Hypertension	1	
Diabetic + Hyperlipidaemia	1	
Hyperlipidaemia + Heartburn	1	
Three medications	10	5.1
Anticoagulant + Hypertension + Hyperlipidaemia	4	
Anticoagulant + Diabetic + Hyperlipidaemia	1	
Anticoagulant + Diabetic + Hypertension	1	
Asthma + Diabetic + Hypertension	1	
Asthma + Anticoagulant + Diabetic	1	
Diabetic + Hyperlipidaemia + Hypertension	1	
Hyperlipidaemia + Anticoagulant + Heartburn	1	
Four medications	4	2.1
Anticoagulant + Diabetic + Hypertension + Heartburn	2	
Anticoagulant + Diabetic + Hypertension + Hyperlipidaemia	1	
Anticoagulant + Hypertension + Heart failure + Hypothyroidism	1	
Other Analgesics		
None	6	3.1
Other Analgesics	189	96.9
One Strong Opioid + One Weak Opioid + One Muscle Relaxant	9	
One Strong Opioid + One Weak Opioid + One Non-Opioid + One Muscle Relaxant + One Anxiolytic	7	
One Strong Opioid + One Weak Opioid + One Anxiolytic	7	
One Strong Opioid + One Muscle Relaxant	7	
One Strong Opioid + One Weak Opioid + One Non-Opioid + One Muscle Relaxant	6	
Two Strong Opioid + One Weak Opioid + One Muscle Relaxant	6	
One Strong Opioid + One Muscle Relaxant + One Anxiolytic	5	
One Weak Opioid + One Anxiolytic	5	
One Weak Opioid alone	5	
One Strong opioid alone	3	
One Anxiolytic alone	2	
Other 80 different combinations (in total)	127	

**Table 3:** Utilization of prescription of nonsteroidal anti-inflammatory drugs (NSAIDs) during admission (*n* = 195).

	Arthroscopy	Reconstructive	Fixation	Total
None	5/108 (4.6%)	0/17 (0%)	1/70 (1.4%)	6/195 (3.1%)
One coxib	64/108 (59.2%)	8/17 (47.1%)	54/70 (71.1%)	125/195 (64.1%)
One tNSAID + One coxib	11/108 (10.2%)	1/17 (5.9%)	4/70 (5.7%)	16/195 (8.2%)
Two coxibs	18/108 (16.7%)	7/17 (41.2%)	8/70 (11.4%)	34/195 (17.4%)
One tNSAID	3/108 (2.8%)	0/17 (0%)	1/70 (1.4%)	4/195 (2.1%)
One tNSAID + Two coxibs	6/108 (5.6%)	1/17 (5.9%)	2/70 (2.8%)	9/195 (4.6%)
Two tNSAIDs + Two coxibs	1/108 (0.9%)	0/17 (0%)	0/70 (0%)	1/195 (0.5%)

Abbreviation: NSAIDs, nonsteroidal anti-inflammatory drugs; coxib, cyclooxygenase-2 inhibitor; tNSAID, traditional nonsteroidal anti-inflammatory drug; +, and.

**Table 4:** Defined daily dose (DDD) indicators and their related variables among types of surgeries.

	Arthroscopy	Reconstructive	Fixation	Overall
Number of inpatients, *n*	108	17	70	195
Average length of stay, day	3.99	6.24	3.79	4.11
Dexketoprofen injection (DDD_WHO_ = 75 mg)				
Number of inpatients receiving drug	5	0	3	8
Total drug prescribed for adult patient (Drug_prescribed_), mg	1100	0	450	1550
Number of DDDs per year (DDD_drug/year_)	14.67	0	6	20.67
DDDs per 100 admissions	13.58	0	8.57	10.60
Ratio of use density to use rate (UD/UR)	73.52	0	52.77	62.85
Diclofenac sodium tablet (DDD_WHO_ = 0.1 g)				
Number of inpatients receiving drug	1	1	1	3
Total drug prescribed for adult patient (Drug_prescribed_), mg	250	50	100	400
Number of DDDs per year (DDD_drug/year_)	2.5	0.5	1	4
DDDs per 100 admissions	2.31	2.94	1.43	2.05
Ratio of use density to use rate (UD/UR)	62.66	8.01	26.39	32.44
Celecoxib capsule (DDD_WHO_ = 0.2 g)				
Number of inpatients receiving drug	21	10	8	39
Total drug prescribed for adult patient (Drug_prescribed_), mg	12,800	7800	5600	26,200
Number of DDDs per year (DDD_drug/year_)	64	39	28	131
DDDs per 100 admissions	59.26	229.41	40.00	67.18
Ratio of use density to use rate (UD/UR)	76.38	62.50	92.35	81.73
Etoricoxib tablet (DDD_WHO_ = 60 mg)				
Number of inpatients receiving drug	10	2	14	26
Total drug prescribed for adult patient (Drug_prescribed_), mg	1710	1350	3720	6780
Number of DDDs per year (DDD_drug/year_)	28.5	22.5	62	113
DDDs per 100 admissions	26.39	132.35	88.57	57.95
Ratio of use density to use rate (UD/UR)	71.43	180.29	116.85	105.75
Parecoxib injection (DDD_WHO_ = 40 mg)				
Number of inpatients receiving drug	99	14	58	171
Total drug prescribed for adult patient (Drug_prescribed_), mg	16,960	2560	10,840	30,360
Number of DDDs per year (DDD_drug/year_)	424	64	271	759
DDDs per 100 admissions	392.59	376.47	387.14	389.23
Ratio of use density to use rate (UD/UR)	107.34	73.26	123.28	108.00

Abbreviation: mg, milligram; DDD, defined daily dose; UD/UR, ratio of use density to use rate.

## Supplementary Materials

The following are available online at https://www.mdpi.com/2226-4787/8/4/235/s1, Table S1: List of selected four types of surgeries, Table S2: Anatomical therapeutic chemical (ATC) code and defined daily dose (DDD) for NSAIDs, Table S3: Interpretation of utilization among types of surgeries.
