# Supplementary material for: Defined Daily Dose and Appropriateness of Clinical Application: The Coxibs and Traditional Nonsteroidal Anti-Inflammatory Drugs for Postoperative Orthopaedics Pain Control in a Private Hospital in Malaysia
Source: Pharmacy (Basel). 2020 Dec 8;8(4):235. doi: 10.3390/pharmacy8040235 (PMC7768540; doi:10.3390/pharmacy8040235)
Supplement: Supplementary file 1 [file pharmacy-08-00235-s001.pdf]

SUPPLEMENT

Supplementary Table 1 List of selected four types of surgeries

**Category 1: Arthroscopy**	**Category 2: Reconstructive**	**Category 3: Fracture Fixation**
• acromioplasty<br>• anterior cruciate ligament<br>• arthroscopy (ankle, elbow, knee, shoulder)<br>• posterior cruciate ligament	• hand replacement<br>• hemiarthroplasty<br>• radial head replacement<br>• shoulder replacement<br>• total hip replacement<br>• total knee replacement	• close manipulative reduction<br>• dynamic hip screw<br>• interlocking nail technique<br>• K. wiring<br>• open reduction internal fixation/plating<br>• tension band wiring

Abbreviation: K. wiring, Kirschner wiring.

Supplementary Table 2 Anatomical therapeutic chemical (ATC) code and defined daily dose (DDD) for NSAIDs

	Generic name	Strength	ATC Code	Route of Administration	Defined Daily Dose (DDD_{WHO})	
					DDD	Unit
tNSAID	Dexketoprofen injection	50mg/2ml	M01AE17	Parenteral	75	mg
	Diclofenac sodium tablet	50mg	M01AB05	Oral	0.1	g
coxib	Celecoxib capsule	200mg, 400mg	M01AH01	Oral	0.2	g
	Etoricoxib tablet	60mg, 90mg, 120mg	M01AH05	Oral	60	mg
	Parecoxib injection	40mg/2ml	M01AH04	Parenteral	40	mg

Abbreviation: ATC, anatomical therapeutic chemical; DDD, defined daily dose; WHO, World Health Organization; mg, milligram; ml, millilitre.

Supplementary Table 3 Interpretation of utilization among types of surgeries

	Arthroscopy	Reconstructive	Fixation	Overall
**Dexketoprofen injection**				
Percentage of patient admitted might get in 2018	13.58	0	8.57	10.60
Appropriateness of clinical application	Yes	NA	Yes	Yes
**Diclofenac sodium tablet injection**				
Percentage of patient admitted might get in 2018	2.31	2.94	1.43	2.05
Appropriateness of clinical application	Yes	Yes	Yes	Yes
**Celecoxib capsule**				
Percentage of patient admitted might get in 2018	59.26	229.41	40.00	67.18
Appropriateness of clinical application	Yes	Yes	Yes	Yes
**Etoricoxib tablet**				
Percentage of patient admitted might get in 2018	26.39	132.35	88.57	57.95
Appropriateness of clinical application	Yes	No	No	No
**Parecoxib injection**				
Percentage of patient admitted might get in 2018	392.59	376.47	387.14	389.23
Appropriateness of clinical application	No	Yes	No	No

Abbreviation: NA, not applicable.
